# Bedside ultrasound training using web-based e-learning and simulation early in the curriculum of residents

**DOI:** 10.1186/s13089-014-0018-9

**Published:** 2015-01-21

**Authors:** Yanick Beaulieu, Réjean Laprise, Pierre Drolet, Robert L Thivierge, Karim Serri, Martin Albert, Alain Lamontagne, Marc Bélliveau, André-Yves Denault, Jean-Victor Patenaude

**Affiliations:** Faculty of Medicine, Department of Medicine and Hôpital Sacré-Coeur, 5400 boul. Gouin ouest, Montréal, H4J 1C5 Canada; Faculty of Medicine, Department of Medicine and Centre d’Apprentissage des Attitudes et Habiletés Cliniques (CAAHC), Université de Montréal, 2900 boul. Edouard-Montpetit, Montréal, H3T 1J4 Canada; Research Centre, Hôpital Sacré-Coeur, 5400 boul. Gouin ouest, Montréal, H4J 1C5 Canada; Faculty of Medicine, Department of Anesthesia, Montreal Heart Institute, Université de Montréal, 5000 Bélanger, Montréal, H1T 1C8 Canada

**Keywords:** Ultrasound, Education, Residents, e-learning, Simulation, Safety

## Abstract

**Background:**

Focused bedside ultrasound is rapidly becoming a standard of care to decrease the risks of complications related to invasive procedures. The purpose of this study was to assess whether adding to the curriculum of junior residents an educational intervention combining web-based e-learning and hands-on training would improve the residents’ proficiency in different clinical applications of bedside ultrasound as compared to using the traditional apprenticeship teaching method alone.

**Methods:**

Junior residents (*n* = 39) were provided with two educational interventions (vascular and pleural ultrasound). Each intervention consisted of a combination of web-based e-learning and bedside hands-on training. Senior residents (*n* = 15) were the traditionally trained group and were not provided with the educational interventions.

**Results:**

After the educational intervention, performance of the junior residents on the practical tests was superior to that of the senior residents. This was true for the vascular assessment (94% ± 5% vs. 68% ± 15%, unpaired student *t* test: *p* < 0.0001, mean difference: 26 (95% CI: 20 to 31)) and even more significant for the pleural assessment (92% ± 9% vs. 57% ± 25%, unpaired student *t* test: *p* < 0.0001, mean difference: 35 (95% CI: 23 to 44)). The junior residents also had a significantly higher success rate in performing ultrasound-guided needle insertion compared to the senior residents for both the transverse (95% vs. 60%, Fisher’s exact test *p* = 0.0048) and longitudinal views (100% vs. 73%, Fisher’s exact test *p* = 0.0055).

**Conclusions:**

Our study demonstrated that a structured curriculum combining web-based education, hands-on training, and simulation integrated early in the training of the junior residents can lead to better proficiency in performing ultrasound-guided techniques compared to the traditional apprenticeship model.

## Background

The use of ultrasound has great potential to immediately provide diagnostic information at the bedside not assessable by physical examination alone. In recent years, there has been an explosion in the number of publications supporting the use of bedside ultrasound in both acute and non-acute care settings [1-3]. Results have shown that its use can speed up and improve the management of a variety of patient conditions and can serve as an invaluable tool for numerous clinical specialties including critical care, emergency medicine, internal medicine, anesthesiology, trauma, and many other disciplines [4-6]. Its use has also been shown to bring frequent changes in diagnosis and subsequent changes in therapy, often resulting in significant improvement in patient care and outcome [7]. But its successful application depends in most part on the user’s skills and is operator-dependent and therein lays one of the greatest challenges faced by clinicians in widely adopting bedside ultrasound. The demand for training is high but all too often, inadequate training programs are in place, if any at all.

The traditional medical education model which uses an apprenticeship approach to clinical learning may need to be re-assessed and augmented with additional training. A competency-based training model may be an important adjuvant to increase clinical skills. Competency-based training can be achieved using simulation, web-based learning tools, and formal hands-on training to allow a clinical procedure to be learned based on a system of standard metrics so that progress and proficiency can be recorded, followed, and assessed [8-11].

An adequate, focused assessment of the vessels and pleural space requires a thorough understanding of ultrasound principles, anatomy, pitfalls, and good interpretation and image acquisition skills. Targeted needle placement under ultrasound guidance requires specific psychomotor skills as well as hand-eye coordination.

The purpose of this study was to assess whether adding to the curriculum of the junior internal medicine residents an educational intervention combining web-based e-learning and hands-on training using human models and simulators would improve the residents’ proficiency in different clinical applications of bedside ultrasound.

## Methods

### Study design

A prospective longitudinal cohort study was used to compare outcomes achieved at specific milestones along the residency program by an intervention and a control group.

### Participants

All residents who started residency in internal medicine at the University of Montreal in July 2008 or after and were still registered as residents at the time of recruitment in the study were eligible to participate. To be included, they had to sign the consent form and agree to study participation requirements. They were excluded if they had studied another profession that uses ultrasound technology (e.g., radiology technician) before entering the residency program.

### Intervention

The participants were trained on the use of bedside ultrasound either using a formal course in addition to the traditional apprenticeship teaching method (intervention group) or using the latter alone (control group).

The course used a combination of self-directed e-learning for acquisition of cognitive knowledge and a series of practical sessions for clinical skills.

Self-directed learning was facilitated using an interactive learning management system (LMS), developed by CAE Healthcare (Montreal, Canada) in collaboration with the Department of Medicine of the University of Montreal. Since 2011, it has been adopted by the American College of Chest Physician (ACCP). It is part of the four mandatory courses leading to the Critical Care Ultrasonography certification. http://www.chestnet.org/Education/Advanced-Clinical-Training/Certificate-of-Completion-Program/Critical-Care-Ultrasonography.

**Figure 1:**
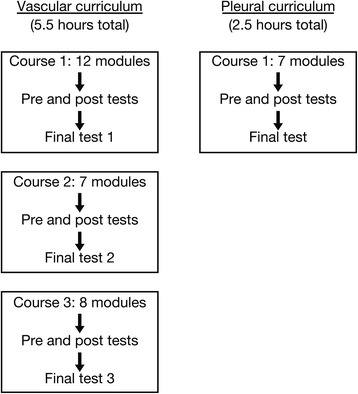
**e-learning curriculum organization.** The vascular (5.5 h duration) and pleural (2.5 h duration) curricula are divided into three and one individual courses, respectively. Each course includes several modules and tests.

This LMS provided descriptions of concepts and clinical applications using multimedia methods such as images, videos, and 3D animations. For each clinical application of bedside ultrasound, the curriculum was divided into one to three individual courses, each one including many modules (Figure 1). In each module, learners assessed their knowledge and learning by completing pre- and post-tests. An average of 10 to 12 h is required to complete the modules (Table 1). Practical sessions (60 to 90 min) consisted of hands-on scanning and clinical teaching at bedside. They were provided by experienced clinical teachers to small groups of participants in various university-affiliated hospitals. Clinical skills were taught using volunteers (sick and healthy) and ultrasound phantoms. During practical sessions, teachers demonstrated how to scan the main vessels and reviewed anatomy, orientation, and pitfalls and how to perform ultrasound-guided needle insertion in various planes. In order to ensure training standardization, clinical teachers were provided with a checklist of items to be covered during each session. Checklists were provided in advance to the participants in order to set expectations and ensure optimal preparation.

**Table 1:** **Summary of the two educational interventions provided to the junior residents group**

**Topic**	**Assessment of central and peripheral vessels**	**Assessment of pleural space and lung**
Cognitive knowledge	Understand basic physical properties of ultrasound	Appreciate general indications for pleural ultrasound and the literature pertinent to it
	Appreciate the literature pertinent to ultrasound-guided vascular access	Understand the general principles of ultrasound examination of the pleural space
	Recognize the criteria for difficult venous access and indications for ultrasound-guided line placement	
	Learn the general principles of ultrasound examination of vessels	
Psychomotor skills	Demonstrate how to perform an ultrasonographic examination of the main vessels in the transverse and longitudinal planes: internal jugular/subclavian/femoral/peripheral vein	Demonstrate how to perform an ultrasonographic examination of the pleural space and lung
Attitudes	Know how to properly interpret and report the ultrasound assessment of vessels and the ultrasound-guided vascular cannulation procedure.	Know how to properly interpret and report the ultrasound assessment of the pleural space and lung and the ultrasound-guided thoracentesis procedure
Self-directed e-learning	5.5 h	2.5 h
Hands-on sessions	5.0 h	2.0 h
Duration	6 weeks	2 weeks

### Study objectives

The study objective is to assess whether adding to the curriculum of the junior internal medicine residents an educational intervention combining self-directed learning and hands-on training improved residents’ competency in different clinical applications of bedside ultrasound as compared to using the traditional apprenticeship teaching method alone.

### Outcome and variables

We used knowledge, skills, and self-confidence as primary endpoints of participants’ competency.

Knowledge was measured as the percentage of accurate responses that the participants provided on a series of comprehensive tests linked to the overall learning objectives of the course. This outcome was assessed using the final tests already integrated in the LMS (Figure 1). These tests were developed based on published guidelines on bedside ultrasound from international expert panel [1,4].

Skills were assessed as the percentage of either accurate responses to questions or well-executed tasks that the participants were requested to perform during practical and oral examinations. Content was based on current guidelines describing clinical knowledge and skills required for bedside ultrasonography [1,4].

An important aspect of the ultrasound assessment is the component related to the guidance of a needle in a specific vessel under direct vision and in real time. This skill was assessed using ultrasound phantoms, both for transverse and longitudinal views, and measured as the success of the guided procedure and the occurrence of posterior wall perforation.

Self-confidence with regard to using bedside ultrasound with real patients was measured on a 5-point Likert scale using a standard question already validated in the literature [12]: *In your future rotations/clinical calls, how confident are you, on a scale of 1 (not at all confident) to 5 (extremely confident), to independently carry out on real patients the tasks reviewed in this examination?*

The participating residents’ socio-demographic characteristics (age, gender, medical school of graduation) and previous exposure to bedside ultrasound were assessed using a self-reported questionnaire.

### Implementation

A convenience sample of residents was assembled after protocol approval by the University of Montreal’s Institutional Review Board (CPER-11-034-D). The internal medicine residency program registration records of the Faculty of Medicine were used to identify the eligible residents. An invitation letter was sent by e-mail to all eligible residents, explaining briefly the project goal and describing in detail the participation requirements and the different aspects of confidentiality, along with the consent form and an invitation to call the study coordinator if they needed more detailed information on the study requirements.

Consenting residents who started residency in 2010 (hereafter referred to as *junior residents*) were assigned to the intervention cohort whereas those who started in the previous 2 years were assigned to the control cohort (*senior residents*). The residents of the intervention cohort were further assigned to the specific clinical teachers that were responsible to carry out the intervention in their clinical setting, i.e., in one of the university-affiliated hospitals. In larger hospitals, the project coordinator randomly allocated the residents between teachers in order to obtain small groups of no more than seven residents.

Clinical teachers were chosen according to the following criteria: recognized expertise in bedside ultrasound and affiliation to the University of Montreal. Since access to LMS data was an integral part of their teaching role, they were aware of test results for the junior residents that were assigned to their own group. However, they were blinded as to which residents had consented to the study, and they did not have access to other data such as those collected by self-report questionnaires nor to any data pertaining to other residents.

As part of their mandatory training, the junior residents were provided with two educational interventions, each covering different areas of bedside ultrasound (assessment of central and peripheral vessels and assessment of pleural space and lung; Table 1). At the onset of the study, the senior residents had had 2 to 3 years of variable clinical exposure to bedside ultrasound and had learned their skills based on the traditional apprenticeship model. The traditional apprenticeship model consists of bedside supervision by the senior residents, critical care fellow, and critical care attendings. Informal courses were provided depending on the critical care staffing interest and expertise. After recruitment in the study, they were not provided with the educational interventions but continued training as usual.

The socio-demographic and previous exposure questionnaire was administered by e-mail at recruitment time and collected by the study coordinator. Knowledge tests were administered online at the end of each intervention, and the participants’ answers were automatically compiled by the LMS software. Skills were measured at baseline for the intervention group and at the end of each intervention for both groups. Practical examinations were administered individually by clinical teachers who were provided with objective assessment criteria and detailed instructions. The six examiners who were used in the intervention group were the same clinical teachers who participated to the study but did not assess their own students. The four examiners used for the control group were not involved in the study. Self-confidence questionnaires were administered following practical skill assessments.

### Statistics

A series of hypotheses were tested by comparing the level of knowledge, skills, and confidence achieved by the participants of the intervention and control cohorts at specific milestones along the residency program, as per protocol. Analyses were conducted with Prism 5.0 (GraphPad Software, La Jolla, CA, USA). Continuous variables were compared with either the Student’s *t* test or the one-way ANOVA followed, when appropriate, with Tukey’s post-tests. Fisher, chi-square, and chi-square for trends tests were used for categorical variables, and Bonferroni correction was applied for multiple comparisons involving the same subset of data. A *p* value <0.05 was considered significant.

## Results

Participant recruitment took place between March 2011 and April 2011, and the study was conducted between May 2011 and September 2011. The participants came from five different hospitals. Thirty-seven junior residents (23 females/14 males: usual gender distribution at the University of Montreal) and 15 seniors (7 females/8 males) went through the different steps of the study. All eligible junior residents from the 2010 internal medicine cohort were recruited. The junior residents were assigned to six clinical teachers according to protocol. The 15 senior residents were recruited on a voluntary basis out of the 70 residents of the 2008 and 2009 internal medicine cohorts.

**Figure 2:**
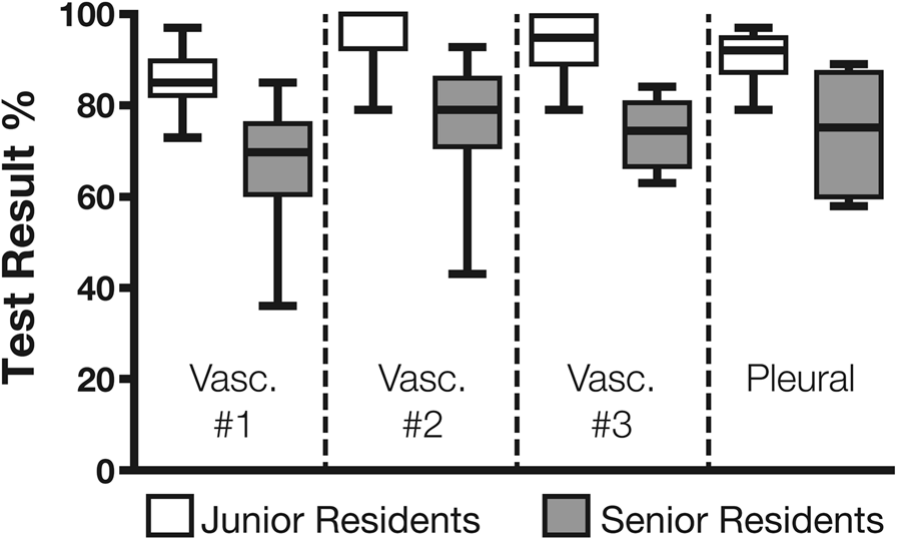
**Vascular and pleural comprehensive test.** Results obtained by the junior and senior residents (median, 25% to 75% interquartile, min.-max.) on the three vascular and one pleural comprehensive online knowledge tests. The junior residents performed better than the seniors for every test (Student’s *t* test: *p* < 0.0001 in all cases).

For the vascular curriculum, the junior residents obtained higher scores than the senior residents on the three knowledge tests (85% ± 6% vs. 67% ± 14%, *p* < 0.0001; 95% ± 7% vs. 77% ± 15%, *p* < 0.0001; 94% ± 8% vs. 74% ± 8%, *p* < 0.0001). Their results were also higher in the case of the pleural and lung curriculum (92% ± 4% vs. 74% ± 12%, *p* < 0.0001) (Figure 2).

**Figure 3:**
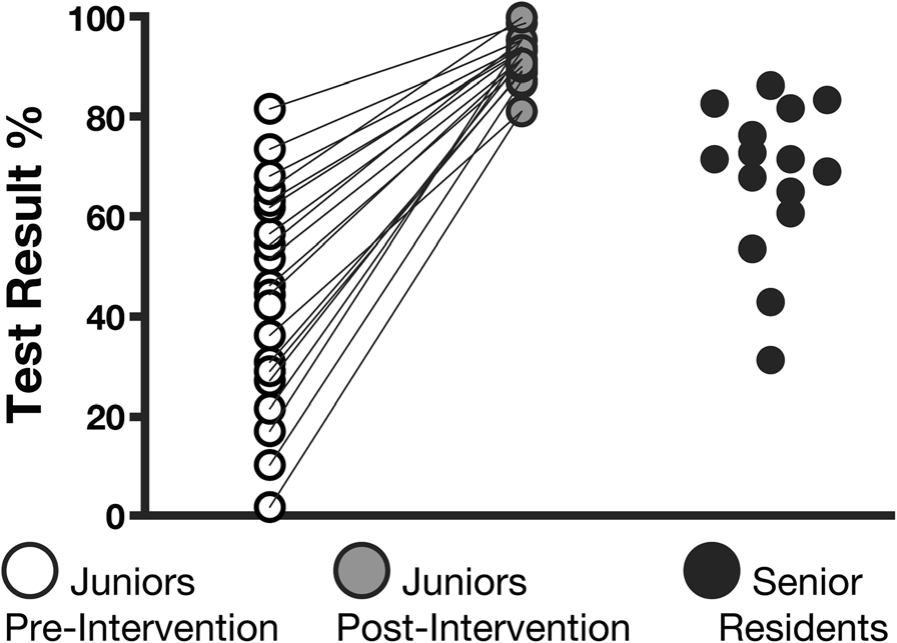
**Vascular practical test.** Performance of the junior and senior residents on the vascular practical test before and after the educational intervention. Significant differences between all groups with one-way ANOVA (*p* < 0.0001) and Tukey’s post-tests: junior post-intervention > senior > junior pre-intervention (*p* < 0.001 for all pairwise comparisons).

**Figure 4:**
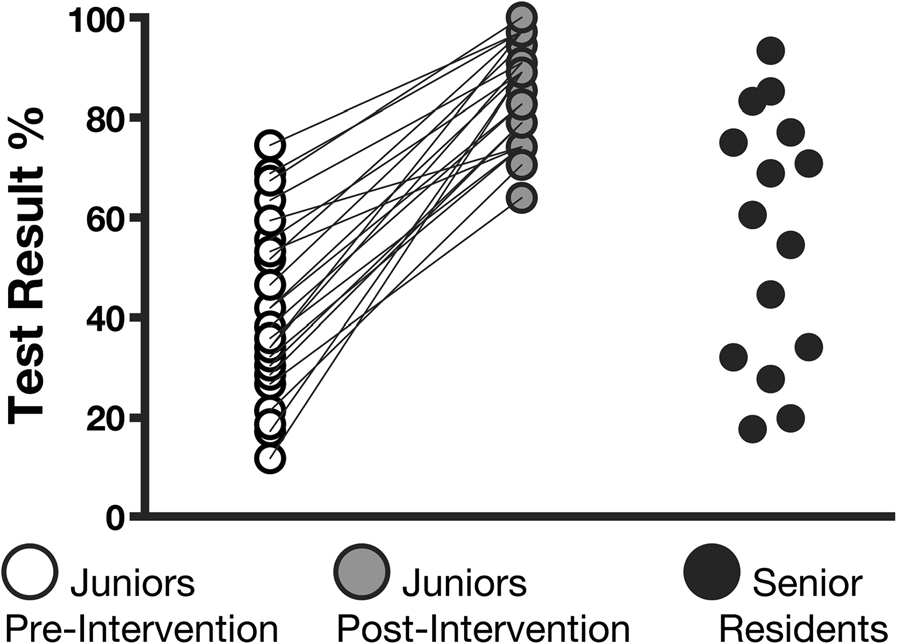
**Pleural practical test.** Performance of the junior and senior residents on the pleural practical test before and after the educational intervention. Significant differences between all groups with one-way ANOVA (*p* < 0.0001) and Tukey’s post-tests: junior post-intervention > senior > junior pre-intervention (junior pre-intervention vs post-intervention *p* < 0.001, junior pre-intervention vs senior *p* < 0.05, junior post-intervention vs. senior *p* < 0.001).

The scores of the junior residents on the practical vascular test significantly improved after the educational intervention (45% ± 20% vs. 94% ± 5%, *p* < 0.001) (Figure 3). The results on the pleural practical test were also improved by the intervention (44% ± 18% vs. 92% ± 9%, *p* < 0.001) (Figure 4).

The results of the senior residents on the vascular (68% ± 15%) and pleural (57% ± 25%) practical tests were lower than those of the junior residents after the educational intervention (*p* < 0.001 in both cases) (Figures 3 and 4).

Before the educational intervention, the success rate of the junior residents in adequately guiding the needle in the vessel was 44% and 56% in the transverse and longitudinal views, respectively (Table 2). In the post-educational intervention assessment, these rates increased significantly to 95% (*p* < 0.0002) and 100% (*p* < 0.0002), respectively. The success rate of the senior residents in both transverse (60%) and longitudinal view (73%) was inferior compared to the results of juniors who benefited from the educational intervention (transverse *p* = 0.0096, longitudinal *p* = 0.0105) (Table 2).

**Table 2:** **Rates for correct needle placement into the vein and accidental perforation of the posterior wall**

	**Junior residents pre-intervention (** ***n*** **= 37)**	**Junior residents post-intervention (** ***n*** **= 37)**	**Senior residents (** ***n*** **= 15)**
View		Transverse - longitudinal	
Success rate in guiding the needle into the vein	44%* to 56%*	95% to 100%	60%** to 73%***
Perforation of the posterior wall of the vein	64%* to 47%****	16% to 14%	27% to 43%

During practical vascular examination. *p < 0.0002, **p = 0.0096, ***p = 0.0105, ****p = 0.0154 vs. junior residents post-intervention (Fisher’s exact test with Bonferroni’s correction for two comparisons).

Before the educational intervention, the rate of perforation of the posterior wall of the vein was 64% and 47% in the transverse and longitudinal views, respectively, for the junior residents. In the post-educational intervention period, these rates decreased to 16% (*p* < 0.0002) and 14% (*p* = 0.0154), respectively. The rates of perforation of the vein’s posterior wall by the senior residents were 27% and 43% in the transverse and longitudinal views, respectively. These results were not significantly different from the results of the juniors who were exposed to the educational intervention (Table 2).

Significant structures were correctly identified more frequently during the pleural space and lung examination by the junior residents following the educational intervention. It was the case for the ribs (pre- vs. post-intervention: 61% vs. 97%, *p* = 0.0002), the diaphragm (37% vs. 97%, *p* < 0.0001), the visceral and parietal pleura (31% vs. 91%, *p* < 0.0001), the normal/consolidated/atelectatic lung (28% vs. 74%, *p* = 0.0001), and the presence of pleural effusion (44% vs. 80%, *p* = 0.0031).

**Figure 5:**
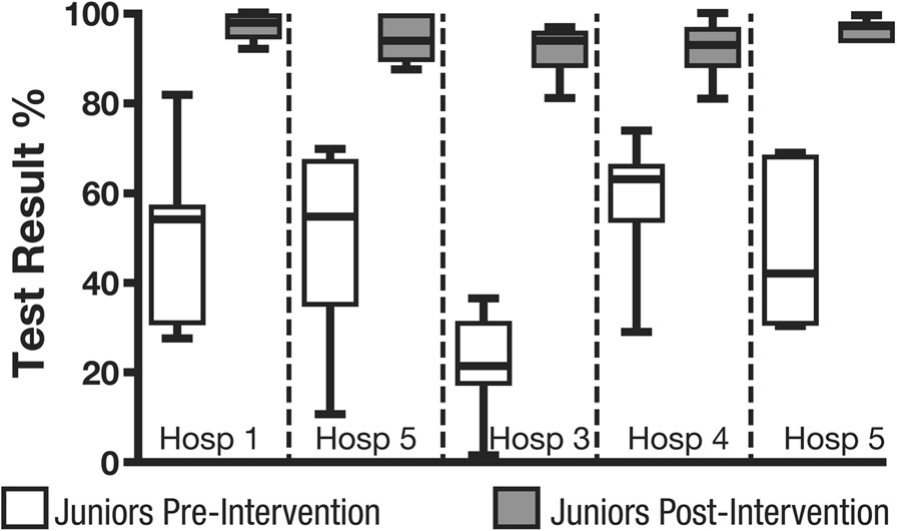
**Educational intervention.** Performance of the junior residents pre- and post-educational intervention (median, 25% to 75% interquartile, min.-max.) in relationship to their source hospital. Pre-intervention scores from hospital 3 were significantly lower (one-way ANOVA *p* = 0.006) than scores from hospitals 1 (*p* < 0.05), 2 (*p* < 0.05), and 4 (*p* < 0.001) (Tukey’s post-tests).

The baseline scores of the junior residents on the practical tests in relationship to the hospital in which they were based were different. We found significant variability for the practical pre-intervention vascular exam (Figure 5). There was no difference between trainees after the intervention in relationship to their affiliated hospital. There was no significant difference either in the results of the comprehensive online vascular or pleural knowledge tests in relationship to the various hospitals in which the residents were based.

No significant relationships were found between the various test scores, gender, and clinical teachers.

Self-confidence was significantly higher amongst the junior residents who completed the educational training than amongst the senior residents (*p* = 0.0093). A total of 45% of the junior residents felt either very or totally confident regarding their ability to perform adequate ultrasound examination compared to 33% of the seniors.

## Discussion

Our study demonstrated that a structured curriculum combining web-based education, hands-on training, and simulation integrated early in the training of the junior residents can lead to better proficiency in performing ultrasound-guided techniques compared to the traditional apprenticeship model.

Some of the very important factors to bedside ultrasound training are to create a valid and standardized training model, ensure that effective learning can occur, and create performance metrics that can accurately test a student’s proficiency that ultimately measures learning [13-15].

When risk is dependent on the level of training, peril is imposed by a traditional training paradigm that involves practicing on patients [8,16-19]. Studies have shown the higher complication rates associated with relative lack of experience in central venous catheter placement [20-23].

Simulation-based education has been used to provide opportunities for safe and deliberate practice, shape the acquisition of clinical skills, and improve patient care and outcomes [10]. Analysis of our results shows a significant improvement in the overall proficiency of the junior residents in performing bedside ultrasound when comparing the pre- and post-educational periods for both the vascular and the pleural space assessment. This improvement occurred over a relatively short period of time of a few months. Also, we have shown a significant improvement in the success rate of the actual ultrasound guidance of the needle in a vessel in both the transverse and longitudinal views when comparing the pre- and post-educational periods. In terms of safety, we have shown that complications related to vascular access using ultrasound-assisted guidance can be reduced as there were fewer posterior wall penetration in the junior resident group after the educational intervention. Posterior wall penetration can be associated with carotid puncture and if too deep other complications such as pneumothorax.

These findings demonstrate the efficacy of the educational intervention in rapidly bringing the junior residents to an adequate level of proficiency by using a blended learning approach. The junior residents enrolled in this study received the educational intervention at the end of their first year - beginning of their second year of residency. It is typically at that time that the residents start doing rotation and taking calls in the intensive care units during which they will often need to proceed with central line insertion and thoracentesis.

From a medical and patient care perspective, improved patient safety is probably one of the most important benefits of having an efficient training program such as the one we have described. Evidence has shown that simulation-based, structured training can not only lead to better proficiency in performing the technique compared to the traditional apprenticeship model but that this better proficiency can also translate into better clinical outcomes. A study by Barsuk et al. [10] who assessed the effect of a simulation-based mastery learning model on central venous catheter insertion skill and the prevalence of procedure-related complications in a medical intensive care unit over a 1-year period showed that the simulation-trained residents had a much higher success rate and fewer needle passes when compared to the traditionally trained residents. More importantly, they showed a dramatic reduction in arterial puncture (1% vs. 14%, *p* < 0.0005) in the simulator-trained group. Another study by Sekiguchi et al. [23] showed a decrease in complications from central line placement in a group of the junior residents who received a structured, pre-rotational training on ultrasound-guided central line placement when compared with previous groups who did not received structured educational training. Placement failure rate decreased from 22.8% to 16.2% (*p* = 0.02), and arterial punctures decreased from 4.2% to 1.5% (*p* = 0.03).

A study by Cohen et al. [24] demonstrated convincingly that simulation-based education in central venous catheter insertion was a cost-effective intervention that directly benefited patients. Specific patient benefits included fewer catheter-related bloodstream infections and decreased length of medical intensive care unit and hospital stays. The simulation-based intervention resulted in saving of more than $800,000, 137 patient hospital days, and around 120 medical intensive care unit days [24]. These studies support the evidence that simulation-based process improvement mechanisms can enhance patient safety and quality of care.

Our standardized blended learning approach using e-learning, hands-on, and simulation offers two important benefits when applied with adequate rigor: (1) it provides a risk-free training environment and (2) it accelerates the learning curve and allows introduction of the procedures on real patients at a safer level of performance. As demonstrated in our study, despite being 1 to 2 years more junior in terms of academic experience and clinical exposure, in the post-educational period, the junior residents were more proficient than the senior residents at performing the overall bedside ultrasound assessment of vessel and pleural space with higher success rate of guided-needle insertion, lower rate of posterior wall perforation, and higher success in identifying the key anatomic landmarks when performing the pleural exam. Although the better scores on both the knowledge and practical tests do not imply the ability to perform the procedure independently, it provides a metric by which educators can ensure a minimum level of proficiency before allowing trainees to perform such procedures on patients under supervision [20]. This reflects a necessary first step in promoting patient safety.

Our educational intervention provides a way to accelerate the learning curve so that it introduces a safer level of performance earlier in the clinical training of the residents.

### Potential limitations

The fact that enrolment of the senior residents was on a voluntary basis could have introduced a form of selection bias in the study as those enrolling themselves in the study might have been those individuals that were more comfortable with bedside ultrasound. This potential bias would potentially reduce the effect of the educational intervention. Despite this, the impact of the educational intervention remained significant. The junior residents performed the practical tests before the educational interventions and after the intervention. One could argue that the junior residents knew what would be asked in the practical test after their training and that this could lead to a better performance. However, the practical tests were very rigorous and geared towards assessing proficiency of the resident in a variety of skills. The various skills tested were rated as being successfully performed only if the examiner felt that the resident had a solid understanding of what was demonstrated. Also, as there were more than 16 weeks separating the initial practical test and the final practical test, it is not likely that the junior residents remembered the questions that were initially asked. Several studies have also shown the benefit of e-learning. Cuca et al. compared traditional versus the e-learning of 75 medical students of a lung ultrasound training program [25]. There were no differences between the groups in terms of retention. Platz et al. studies physicians from two German emergency departments who were randomized into a classroom group with traditional lectures and a web group who watched narrated lectures online [26]. All participants completed a pre- and post-test and a second post-test 8 weeks later. Both the classroom and web group showed significant improvement in pre- and post-test scores with no differences in terms of retention at 8 weeks. Finally, another study by Blackstock et al. evaluated sonographic knowledge in 45 medical students using web-based tutorials. They found similar results compared to an emergency medicine resident [27]. The major difference between these studies and ours is that both theoretical knowledge and clinical skills were also assessed in terms of vascular access and pleural examination.

## Conclusions

Our study demonstrated that a structured curriculum combining web-based education, hands-on training, and simulation can lead to better proficiency in performing ultrasound-guided techniques compared to the traditional apprenticeship model. Future studies are necessary to demonstrate that this better proficiency will translate into better clinical outcomes and overall improved patient care.
